# Comment on “Predicting the mortality due to Covid-19 by the next month for Italy, Iran and South Korea; a simulation study” 

**Published:** 2020

**Authors:** Samira Chaibakhsh, Mohsen Vahedi

**Affiliations:** 1 *Eye Research Center, The five Senses Institute, Rassoul Akram Hospital, Iran University of Medical Sciences, Tehran, Iran*; 2 *Department of Biostatistics, University of Social Welfare and Rehabilitation Sciences, Tehran, Iran *

## To The Editor

 We have read with great interest the manuscript by Shojaei et al (1) who used statistical models to predict the mortality due to Covid-19 for Italy, Iran, and South Korea for the next month from 3/15/2020. It was claimed that the distribution of mortality was Poisson, so the corresponding methods were used to perform simulations (2). Now, the actual values of mortality have been released by the WHO for the prediction period, and the simulations showed an acceptable prediction; however, we would like to make some comments.

**Figure 1:**
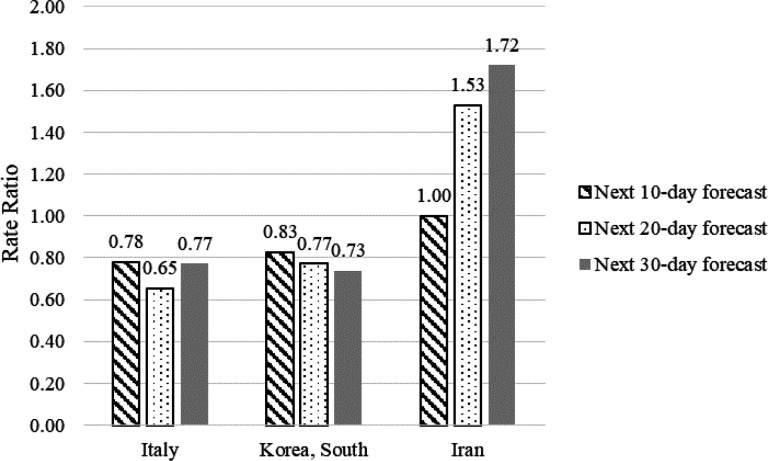
Evaluation of predictions in three countries in forecasting intervals using Rate Ratio

Figure 1 shows the rate ratios (actual rate/prediction rate) in Italy, South Korea, and Iran. The rate ratios showed an overestimation of the mortality rate in Italy (0.78, 0.65, 0.77) and South Korea (0.83, 0.77, 0.83), but an underestimation in Iran (1, 1.53, 1.72). The best predictions, as expected, were due to the next 10-day forecast. Although the same pattern of errors in predictions occurred for Italy and South Korea, the relative bias (3) of number of deaths (-0.29) and confirmed cases (-0.04) shows that the overestimation of the mortality rate in Italy was due to the underestimation of the number of deaths. In South Korea, however, the relative bias showed that overestimations were the result of overestimated confirmed cases (0.48), not the underestimation of number of deaths (0.14). 

Relative ratios showed that predictions in Iran were lower than the actual reports (Figure 1). The relative bias of number of deaths was remarkably underestimated (0.35), but the number of confirmed cases was similar to the actual number (-0.03). 

All three models utilized the same scenario, yet the results of predictions for Iran differed. Thus, it seems that the number of deaths due to Covid-19 in Iran was reported to be lower. The predicted number of confirmed cases was very acceptable; thus, the hypothesis that the reported number of deaths from Iran was lower than the actual number becomes stronger. 

## Conflict of interests

The authors declare that they have no conflict of interest.

## References

[1] Shojaee S, Pourhoseingholi MA, Ashtari S, Vahedian-Azimi A, Asadzadeh-Aghdaei H, Zali MR (2020). Predicting the mortality due to Covid-19 by the next month for Italy, Iran and South Korea; a simulation study. Gastroenterol Hepatol Bed Bench.

[2] Kutner MH, Nachtsheim CJ, Neter J, Li W (2005). Applied linear statistical models.

[3] Rothman AJ, Klein WM, Weinstein ND (1996). Absolute and Relative Biases in Estimations of Personal Risk. J Appl Soc Psychol.

